# Like a bridge over troubled water? A longitudinal study of general social support, colleague support, and leader support as recovery factors after a traumatic event

**DOI:** 10.1080/20008198.2017.1302692

**Published:** 2017-03-20

**Authors:** Marianne Skogbrott Birkeland, Morten Birkeland Nielsen, Marianne Bang Hansen, Stein Knardahl, Trond Heir

**Affiliations:** ^a^ Norwegian Centre for Violence and Traumatic Stress Studies, Oslo, Norway; ^b^ National Institute of Occupational Health, Oslo, Norway; ^c^ Department of Psychosocial Science, University of Bergen, Bergen, Norway; ^d^ Institute of Clinical Medicine, Faculty of Medicine, University of Oslo, Oslo, Norway

**Keywords:** Trauma, terrorism, psychological distress, growth curve analysis, LGM

## Abstract

**Background**: Whereas the association between social support and psychological distress has been well-established through both cross-sectional and longitudinal studies, less is known about whether social support influences rate of change in psychological distress over time. Nor is it clear whether social support predicts baseline psychological distress, or, more importantly, whether social support may contribute to more rapid recovery following trauma exposure.

**Objective**: This study aimed to determine the extent to which social support contributed to the recovery process among individuals with psychological distress after being exposed to trauma.

**Methods**: Prospective survey data from ministry employees were collected 10, 22, and 34 months after the 2011 Oslo bombing that targeted the governmental quarters. We explored recovery in a clinical subsample (N = 238) of individuals with elevated levels of psychological distress (defined as mean 10-item Hopkins symptom checklist score > 1.85) one year after the event. A linear latent growth curve of psychological distress with general social support from friends and family, colleague support, and leader support as predictors was examined.

**Results**: High levels of general social support and leader support were independently associated with a more rapid decline in psychological distress over time.

**Conclusions**: General social support, as well as support from a leader in one’s working life, may facilitate recovery from psychological distress after exposure to a traumatic event. Enhancing social support from family and friends, as well as in work settings, may benefit those with psychological distress following a traumatic workplace event.

## 1. Introduction

Experiencing a traumatic event, such as a terrorist attack targeting one’s workplace, may lead to elevated levels of psychological distress (Neria, DiGrande, & Adams, 2011). Although previous studies have established that a low level of social support is among the strongest predictors of posttraumatic stress (Brewin, Andrews, & Valentine, 2000; Ozer, Best, Lipsey, & Weiss, 2003; Pietrzak, Johnson, Goldstein, Malley, & Southwick, 2009), there is a shortage of prospective studies examining whether social support influences recovery or rate of change in psychological distress over time.

Social support has been defined as instrumental, informational, and emotional support (House & Kahn, 1985). Instrumental support refers to behavioural or material help with practical tasks or problems. Informational support consists of the provision of information or advice that may help in problem-solving, and may also include feedback on a person’s interpretation of a situation and guidance on how to proceed with further action. Emotional support is the demonstration of love and caring, encouragement, and sympathy. These functions may be generally helpful and thus have a main effect on psychological distress regardless of exposure to trauma. In line with this, a review concluded that high perceived emotional and instrumental support, as well as having a large and diverse support network, is associated with low levels of depression (Santini, Koyanagi, Tyrovolas, Mason, & Haro, 2015).

In addition, social support may have a protective effect on health in adverse circumstances, such as after experiencing a traumatic event (Cohen & Wills, 1985). As such, social support had a buffering effect on the relationship between exposure severity and general distress among tourists after the 2004 Indian Ocean tsunami (Arnberg, Hultman, Michel, & Lundin, 2012). Furthermore, for individuals struggling with high levels of psychological distress, social support may act as a changing agent. For example, several studies examining the effects of social support on later posttraumatic stress have found that high perceived general social support is associated with lower subsequent levels of posttraumatic stress (Freedman, Gilad, Ankri, Roziner, & Shalev, 2015; Kaniasty & Norris, 2008; Shallcross, Arbisi, Polusny, Kramer, & Erbes, 2016).

Social support may be especially important after intentional traumatic events such as a terrorist attack, compared to after non-intentional traumatic events. Intentional traumatic events are associated with worse health outcomes than inadvertent harm (Santiago et al., 2013). As terrorists’ main goals are to generate fear, terror, intimidation, and mistrust (Rudenstine & Galea, 2015), social support may have a special function for recovery of feelings of trust and safety. This is relevant both for individuals who are directly exposed to the terrorist attack, and for individuals who may not be present at the site of the terrorist attack, but who are also at risk of developing psychological distress (Hansen et al., in press; May & Wisco, 2015; Schlenger et al., 2002).

Sources of social support other than family and friends may also contribute to lower psychological distress. For example, cross-sectional studies show that a low level of social support in the workplace is associated with psychological distress when controlling for both micro- and macro-level predictors (Marchand & Blanc, 2011; Marchand, Demers, & Durand, 2005, 2006). Prospective studies of the relationship between a broad set of work factors and psychological distress among employees across a wide variety of organizations have established that low leader support is one of the most consistent predictors of psychological distress (Finne, Christensen, & Knardahl, 2014; Nielsen, Tvedt, & Matthiesen, 2012).

Different sources of social support may have overlapping or independent effects on psychological distress. One possibility is that self-reported social support from friends, family, colleagues, and leaders reflects one common factor of psychological capital (Li et al., 2014), and that they may serve similar functions in trauma recovery. Another possibility is that the sources of social support have different functions, and may be differentially associated with psychological distress (Walen & Lachman, 2000). For example, support from friends and family may be crucial for recovery from psychological distress, whereas the effect of support from leaders and colleagues may be negligible. Yet another possibility is that support from different sources has additive and independent effects on psychological distress.

Whereas the association between social support and psychological distress is well-established in both cross-sectional and longitudinal studies, within both trauma and work environment contexts, less is known about whether social support influence the rate of change in psychological distress over time. It is unclear whether social support only predicts baseline levels of psychological distress, or, more importantly, whether social support may also contribute to more rapid recovery after trauma exposure. This information is important to clinicians and others who work on strategies for interventions and preventive measures after trauma exposure in the workplaces as well as other contexts.

High levels of social support may be associated with low levels of psychological distress at baseline, but cannot be expected to be associated with further decline in psychological distress within this group that already reports low psychological distress. Still, in the subgroup with high baseline levels of psychological distress, high levels of social support may be associated with faster decline in psychological distress. This is called Simpson’s paradox: inferences drawn at the population level may not apply to subgroups with different starting levels or degrees of stability over time (Kievit, Frankenhuis, Waldorp, & Borsboom, 2013). Therefore, for the present study, we chose to focus on individuals with high baseline levels of psychological distress.

This three-wave longitudinal study aimed to determine the extent to which social support from leaders, colleagues, family and friends contributes to the recovery process among individuals with psychological distress after being exposed to trauma, over a three-year time period. We focused on individuals with high baseline levels of psychological distress, and whether – and to what extent – general social support from family and friends, colleague support, and leader support may facilitate recovery from psychological distress after trauma. Specifically, we hypothesized, on the basis of previous cross-sectional and time-lagged findings of negative relationships between support and distress, that high levels of general social support from family and friends, colleague support, and leader support would be independently associated with a decline in psychological distress over time.

## 2. Methods

### 2.1. Sample and design

This prospective three-wave study with full-panel design used a sample of ministerial employees after the 2011 Oslo bombing attack. This terror attack was directed at the Norwegian government. A car bomb explosion in the executive governmental quarter of the city centre shattered governmental buildings, killed eight people and injured 209 others. All employees in 14 of the 17 Norwegian ministries were invited to participate in the research project ‘Mental health and work environment factors in the aftermath of the Oslo terrorist attack July 22nd, 2011’ (Hansen et al., in press; Hansen, Nissen, & Heir, 2013). Data were collected 10, 22 and 34 months after the bombing, in April/May 2012 (T1), in April/May 2013 (T2), and in April/May 2014 (T3). Of the 3520 eligible employees, 1972 (56%) responded at T1 (838 men, 1134 women), 1780 (51%) at T2 (737 men, 1043 women), and 1580 (45%) at T3 (688 men, 892 women). Participants with low baseline levels of psychological distress at T1 measured by the 10-item Hopkins symptom checklist (HSCL-10 < 1.85; Strand, Dalgard, Tambs, & Rognerud, 2003) were excluded, resulting in a sample of N = 238 individuals (14% of the total sample; 67 men, 171 women) who experienced psychological distress at T1. Strict procedures were followed to ensure confidentiality and the study was approved by the regional committees for medical and health research ethics.

### 2.2. Measures

Psychological distress was measured using the HSCL-10, which measures depression- and anxiety-related symptoms. Item examples include ‘Suddenly scared for no reason’, ‘Feeling tense or keyed up’, and ‘Feeling of worthlessness’. Respondents indicated the relevance of each symptom from ‘Have not experienced this symptom’ (1) to ‘Have experienced this symptom a lot’ (4). As proposed by Strand and colleagues, the cut-off for ‘caseness’ was a mean score ≥1.85 (Strand et al., 2003). Cronbach’s alphas were 0.76, 0.87, and 0.89 at T1, T2, and T3, respectively.

General social support was measured with four items from the crisis support scale (CSS) (Elklit, Pedersen, & Jind, 2001). These items (‘Someone willing to listen’, ‘Able to talk about thoughts and feelings’, ‘Sympathy and support from others’, and ‘Practical help’) are categorized as positive social support. Replies were measured on a seven-point scale, ranging from ‘Never’ (1) to ‘Always’ (7), and were averaged. Cronbach’s alphas were 0.89, 0.91, and 0.91 at T1, T2, and T3, respectively.

Leader and colleague support was measured with scales from the general Nordic questionnaire for psychological and social factors at work (QPS Nordic) (Dallner et al., 2000). The items include: ‘If needed, can you get support and help with your work from your immediate superior?’, ‘If needed, is your immediate superior willing to listen to your work-related problems?’, and ‘Are your work achievements appreciated by your immediate superior?’ (i.e. leader support); and ‘If needed, can you get support and help with your work from your colleagues?’ and ‘If needed, are your coworkers willing to listen to your work-related problems?’ (i.e. colleague support). Response categories ranged from ‘Very seldom’ (1) to ‘Very often or always’ (5). Cronbach’s alphas were: 0.85, 0.90, and 0.86 for leader support and 0.76, 0.81, and 0.78 for colleague support at T1, T2, and T3, respectively.

Exposure to the actual site or epicentre of the explosion was assessed by asking employees where they were located when the bomb went off, using five exposure categories: (1) ‘in the government district downtown’, (2) ‘in downtown Oslo, but not in the government district’, (3) ‘in Oslo, but not downtown’, (4) ‘in Norway, but not in Oslo’ and (5) ‘abroad’. These categories were subsequently collapsed into two categories (1 and 2–5) reflecting direct (coded as 1) and indirect exposure (coded as 0). We also asked whether participants had witnessed dead or dying people; whether they had witnessed people seriously injured; whether they had been physically injured themselves, whether they had experienced that a close colleague were physically injured or died, and whether their office had been damaged (see Table 1).

**Table 1.** Characteristics of the sample.

	N ≈ 238
Age (years) M ± SD	45.1 (10.8)
Gender (female %)	72
Education (low/mid/high %)	15 /27 /57
Present during bomb explosion (yes %)	22
Did you witness dead/dying people? (yes %)	8
Did you witness seriously injured people? (yes %)	18
Were you injured? (yes %)	8
Was a colleague injured? (yes %)	62
Did a colleague of yours die? (yes %)	24
Office damage? (yes %)	68
Other traumatic event last year before T1 (yes %)	9
Other traumatic event between T1 and T2 (yes %)	8
Other traumatic event between T2 and T3 (yes %)	6

Information about experiencing other traumatic events in the 12 month period prior to each of the three waves of data collections was obtained by the question ‘Have you experienced or witnessed other serious incidents (e.g. natural disaster, serious accident, violence, robbery or assault) during the last 12 months?’ and coded as 0 (no) or 1 (yes).

### 2.3. Statistical analyses

We used structural equation modelling (SEM) to analyse the relationships between leadership and psychological distress. SEM analyses were conducted in four steps. In the first step, we used confirmatory factor analyses to examine the measurement models as well as test for dimensionality of three latent variables of general social support, leader support and colleague support. Second, we specified unconditional latent growth curve models (LGM) of psychological distress, general social support, leader support, and colleague support. LGM assumes heterogeneity in both initial status (intercept) and change over time (slope). By capturing individual differences in intercept and slope, LGM makes it possible to study relationships between predictors and change over time (Duncan, Duncan, & Strycker, 2013). Third, we used a conditional growth curve model with intercepts and slopes of psychological distress regressed on sex, exposure, general social support, leader support, and colleague support measured at T1. In these analyses, all continuous variables were centralized and brought into the model at the same time, and the constructs were adjusted for each other (Model 1). Finally, we re-specified the conditional growth curve so that the intercept reflected the end point (T3), and regressed this second set of intercept and slope on the same predictors (Model 2).

All data modelling was performed with Mplus Version 7.2 (Muthén & Muthén, 1998–2014). To correct for somewhat skewed distributions, maximum likelihood estimation with robust errors (MLR) was applied. We assessed chi-squared (χ^2^), root mean square error of approximation (RMSEA), comparative fit index (CFI), and standardized root mean square residual (SRMR) to determine model fit. Values of RMSEA <0.05 denote a well-fitting model (Hu & Bentler, 1999), but it is known that RMSEA does poorly when N is low (Kenny, Kaniskan, & McCoach, 2014). CFI >0.95 indicates a well-fitting model, and >0.90 indicates an acceptable model (Kline, 2011). Values of SRMR that are acceptable generally follow the same guidelines for interpreting the RMSEA (Little, 2013).

### 2.4. Missing data

Of the 238 participants at T1, 172 responded at T2 and 171 responded at T3; 134 responded at both T2 and T3; 38 responded only at T1 and 37 only at T2. Level of psychological distress at T1 was not significantly associated with probability of responding at T2 (odds ratio: 1.90, *p *= 0.066) or T3 (odds ratio: 1.00, *p *= 0.993). Similarly, the level of psychological distress at T2 was not associated with the probability of responding at T3 (odds ratio: 0.96, *p *= 0.902). Thus, missingness was not significantly related to the main variables of interest, a situation consistent with missing completely at random (MCAR). However, because these results may have occurred due to low sample size and low power, we assumed a less restrictive condition: missing at random (MAR). Thus, the Mplus 7.2 inbuilt full information maximum likelihood (FIML) estimation with robust standard errors was used to handle missing data. This approach assumes data are MAR, and all observed information is used to produce the maximum likelihood estimation of parameters. This is one of the best approaches currently available for handling missing data (Graham, 2009).

## 3. Results

### 3.1. Sample characteristics

In order to study how social support relates to recovery from psychological distress, we analysed data from a subsample of 238 individuals who were characterized by high baseline psychological distress, according to common criteria (Strand et al., 2003). Compared to individuals with no or low levels of baseline psychological distress, our subsample consisted of a higher percentage of women [χ^2^(1, 1923) = 22.21, *p *< 0.001], and a higher percentage with high levels of education [χ^2^(1, 1922) = 8.43, *p *= 0.015]. They did not differ according to age [F(1,1922) = 0.22, *p *= 0.643], but were more severely exposed to the bomb attack than individuals with low levels of psychologically distress [F(1,1923) = 47.97, *p *< 0.001]. Compared to individuals experiencing no or low baseline levels of psychological distress, on average the distressed subsample reported significantly lower baseline levels of general social support [F(1,1895) = 183.92, *p *< 0.001], leader support [F(1,1789) = 125.23, *p *< 0.001], and colleague support [F(1,1785) = 84.72, *p *< 0.001].

Seventy-two per cent of our sample were women, 22% were present in the buildings during the bomb attack, 8% were injured themselves, 62% reported having an injured colleague, and 68% experienced that their office was damaged (see Table 1 for sample characteristics). Mean levels of psychological distress seemed to decrease over time, whereas means of all types of social support seem to increase from 10 months (T1) to three years (T3) after the bomb attack (see Table 2). Notably, the magnitudes of the negative correlations between all sources of social support and psychological distress measured at the same time seem to increase over time (e.g. correlations between leader support and psychological distress were −0.02, *p *= 0.823 at T1, and −0.30, *p *= 0.001 at T3).

**Table 2.** Means (SD), and correlations between psychological distress, exposure, and different types of support, t1 ≤ 1.85 (N ≈ 238).

	Range	Mean (SD)	1	2	3	4	5	6	7	8	9	10	11	12	13	14	15
1. Psychological distress T1	1–4	2.38 (0.46)															
2. Psychological distress T2	1–4	2.10 (0.60)	0.48**														
3. Psychological distress T3	1–4	1.99 (0.62)	0.43**	0.67**													
4. Direct exposure	0–1	0.22 (0.42)	0.12	0.15													
5. Other traumas T1	0–1	0.09 (0.29)	0.05	0.03	0.04	−0.03											
6. Other traumas T2	0–1	0.08 (0.27)	0.00	0.05	0.12	0.08	0.19*										
7. Other traumas T3	0–1	0.06 (0.23)	0.05	0.02	0.11	0.02	0.11	0.35**									
8. Social support T1	1–7	4.52 (1.45)	−0.11	−0.21**	−0.17*	0.05	−0.02	−0.05	−0.13								
9. Social support T2	1–7	4.46 (1.53)	0.01	−0.31**	−0.27**	0.05	−0.08	−0.05	−0.12	0.66**							
10. Social support T3	1–7	4.70 (1.49)	−0.08	−0.23**	−0.32**	0.02	−0.09	−0.12	−0.17*	0.61**	0.72**						
11. Colleague support T1	1–5	3.61 (0.84)	−0.02	−0.13	−0.04	−0.05	0.04	−0.06	0.03	0.51**	0.36**	0.37**					
12. Colleague support T2	1–5	3.65 (0.84)	0.06	−0.20*	−0.15	0.19*	−0.02	−0.02	−0.05	0.30**	0.41**	0.32**	0.50**				
13. Colleague support T3	1–5	3.84 (0.82)	−0.04	−0.22*	−0.28**	0.03	−0.03	−0.23*	0.01	0.33**	0.46**	0.47**	0.52**	0.48**			
14. Leader support T1	1–5	3.34 (0.93)	−0.02	−0.13	−0.13	−0.01	−0.13	−0.13	−0.07	0.30**	0.24**	0.23**	0.48**	0.29**	0.32**		
15. Leader support T2	1–5	3.49 (0.95)	−0.04	−0.32**	−0.24*	0.11	−0.13	−0.04	−0.08	0.26**	0.36**	0.22*	0.30**	0.52**	0.30**	0.52**	
16. Leader support T3	1–5	3.62 (0.97)	−0.21*	−0.24*	−0.30**	0.06	0.00	−0.14	−0.05	0.18*	0.28**	0.32**	0.28**	0.31**	0.54**	0.54**	0.58**

* *p *< 0.05, ** *p *< 0.01, *** *p *< 0.001

### 3.2. Measurement models

To assess the measurement models and dimensionality of social support factors, a series of confirmatory factor analyses was conducted. A model with three correlated factors of social support at T1 was superior to models with one or two correlated factors at both time points [χ^2^ (24, N = 237) = 97.535, *p *< 0.001, CFI = 0.925, RMSEA = 0.114]. Inspection of the modification indices suggested a local dependency between one of the leader support items (‘If needed, can you get support and help with your work from your immediate superior?’) and one of the colleague support items (‘If needed, can you get support and help with your work from your colleagues?’); allowing these items to be correlated increased the model fit to very good [χ^2^ (23, N = 237) = 47.318, *p *< 0.001, CFI = 0.975, RMSEA = 0.067]. However, for conceptual reasons the original scales were kept and additional analyses used observed mean scores levels for the scales.

### 3.3. Change over time

The unconditional latent linear growth curve model for psychological distress provided an acceptable model fit, whereas model fit for the unconditional latent linear growth curve models of general social support, colleague support, and leader support was excellent (see Table 3). The unstandardized estimates of means of intercepts and slopes show that on average, psychological distress decreased across time, whereas support from colleagues and leaders increased. Furthermore, on average, general social support was stable over time. The variance estimates indicate that there were considerable individual differences in baseline levels, and change over time, of psychological distress. Because there were no significant individual differences in the rate of change of any of the social support constructs, we decided to use only the baseline levels of these constructs in further analyses.

**Table 3.** Unstandardized parameter estimates and standard errors (SE), and model fit indices for latent growth curve models of psychological distress and social support.

	Psychological distress			General social support			Colleague support			Leader support		
	Estimate	SE	*p*	Estimate	SE	*p*	Estimate	SE	*p*	Estimate	SE	*p*
*Means*												
Intercept	2.36	0.03	0.000	4.52	0.09	0.000	3.60	0.06	0.000	3.34	0.06	0.000
Slope	−0.19	0.02	0.000	0.06	0.05	0.202	0.10	0.03	0.004	0.14	0.04	0.000
*Variances*												
Intercept	0.12	0.04		1.40	0.25		0.33	0.05	0.000	0.49	0.06	0.000
Slope	0.06	0.02	0.003	0.15	0.12	0.000	-	-		-	-	
*Covariance*	0.00	0.03	0.011	−0.02	0.14	0.216	-	-		-	-	
χ^2^	8.099			0.186			1.135			2.076		
RMSEA	0.173			0.000			0.000			0.000		
CFI	0.930			1.000			1.000			1.000		
SRMR	0.044			0.005			0.038			0.067		

### 3.4. Prediction of baseline and rate of change

In order to test whether general social support, colleague support, and leader support at T1 were associated with baseline and rate of change in psychological distress, we regressed intercept and slope of psychological distress on general social support, colleague support, leader support, exposure, and sex (see Table 4, model 1). Because the percentage who reported having experienced other traumatic events was low, and having experienced them was not associated with psychological distress in this sample (see Tables 1 and 2), these variables were not included in the analyses. The estimates show that when controlling for direct exposure and sex, high general social support, but not support from colleagues or leaders, was associated with low baseline levels of psychological distress (−0.20, *p *= 0.045). Moreover, the estimates show that high levels of general social support and leader support were independently associated with a steeper decline in psychological distress (−0.21, *p *= 0.050, and –0.21, *p *= 0.055, respectively). Direct exposure, sex or colleague support were not associated with the rate of change in psychological distress. At our end point (three years after the incident), direct exposure and low general social support were still significantly associated with high levels of psychological distress, whereas sex, colleague support, and leader support were not independently associated with levels of psychological distress.

**Table 4.** Standardized estimates and standard errors (SE) of associations between intercepts (I) and slopes (S) of psychological distress, and direct exposure, sex, and sources of social support measured at T1.

	Model 1: Intercept parameterized as baseline status (10 months after)						Model 2: Intercept parameterized as endpoint (three years after)					
	I on			S on			I on			S on		
	Est	SE	p	Est	SE	p	Est	SE	p	Est	SE	p
Direct exposure	0.16	0.09	0.089	0.09	0.09	0.271	0.17	0.07	0.016	0.09	0.09	0.271
Sex	0.21	0.07	0.006	−0.17	0.09	0.055	−0.02	0.08	0.804	−0.17	0.09	0.055
General social support	−0.20	0.10	0.045	−0.21	0.11	0.050	−0.29	0.09	0.001	−0.21	0.11	0.050
Colleague support	0.04	0.08	0.643	0.11	0.12	0.391	0.11	0.11	0.281	0.11	0.12	0.391
Leader support	0.02	0.09	0.841	−0.21	0.11	0.048	−0.16	0.09	0.065	−0.21	0.11	0.048
χ^2^	8.710						8.710					
RMSEA	0.045						0.045					
CFI	0.980						0.980					
SRMR	0.024						0.024					

**Figure 1.**
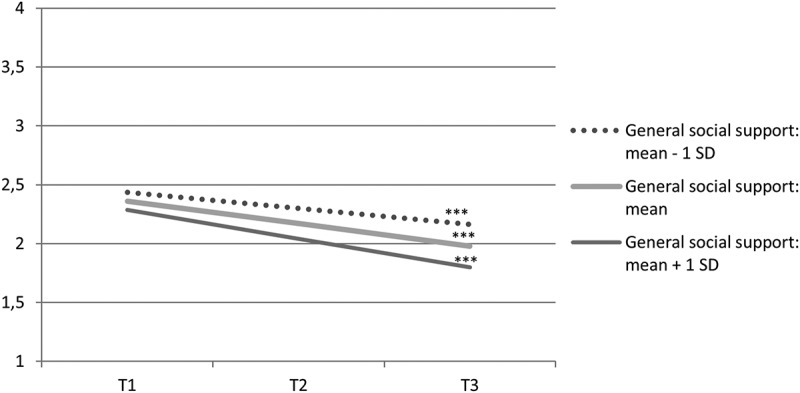
Estimated mean development of psychological distress from 10 months (T1) to three years (T3) after the 2011 Oslo bombing, dependent on level of general social support, when exposure, sex, colleague support, and leader support are controlled for. *** slope: *p *< 0.001.

**Figure 2.**
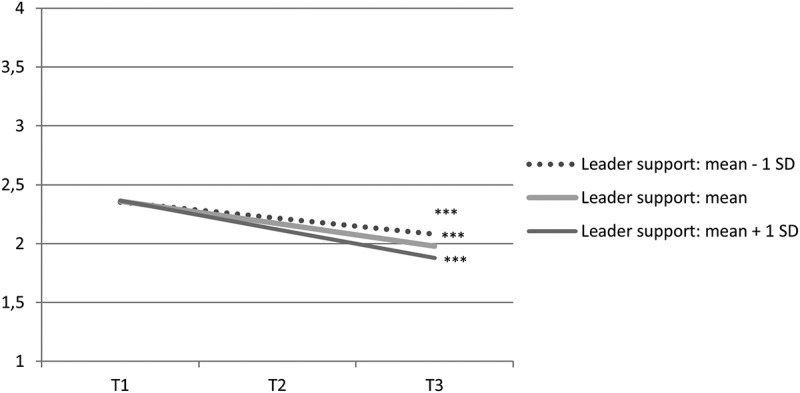
Estimated mean development of psychological distress from 10 months (T1) to three years (T3) after the 2011 Oslo bombing, dependent on level of leader support, when exposure, sex, colleague support, and general social support are controlled for. *** slope: *p *< 0.001.

The relationships between predictors and slopes were further probed by calculating slopes of psychological distress at different levels of predictors individually (Curran, Bauer, & Willoughby, 2004). By obtaining point estimates for intercept and slopes on selected predictor values, we tested whether the effect of time was different across levels of the predictor when controlled for other predictors in the model. Figures 1 and 2 show how slopes of psychological distress were dependent on general social support and leader support. The estimated slopes of psychological distress for those reporting general social support one standard deviation below the mean, at mean, and one standard deviation above the mean were −0.14 (*p *< 0.001), −0.19 (*p *< 0.001), and −0.25 (*p *< 0.001), respectively. The estimated slopes of psychological distress for those reporting leader support at one standard deviation below the mean, at mean, and one standard deviation above the mean were −0.14 (*p *= 0.007), −0.19 (*p *< 0.001), and −0.25 (*p *< 0.001), respectively. This indicates that on average, levels of psychological distress declined significantly for most individuals, but the decline occurred more rapidly for those with higher leader and general social support.

## 4. Discussion

Expanding on previous cross-sectional studies, the results of this study show that higher levels of general social support and support from leaders are associated with a more rapid decline in psychological distress over time. Moreover, support from colleagues was not significantly associated with baseline levels or rate of change of psychological distress over time when controlling for other sources of support.

In this group of initially psychologically distressed employees, the average levels of psychological distress decreased over time. Simultaneously, perceived support from colleagues and leaders increased, whereas average perceived general social support from friends and family was stable over time. This stands in contrast to previous studies showing that early psychological distress after trauma (e.g. posttraumatic stress) is related to a decline in perceived social support over time (King, Taft, King, Hammond, & Stone, 2006; Lui, Glynn, & Shetty, 2009; Price, Evans, & Bagrow, 2014; Wu & Cheung, 2006). On the contrary, the present sample reported increasing levels of perceived support from colleagues and leaders. This suggests that these employees may have experienced a mobilization of support and an increased sense of community within their workplace. Additionally, the terror attack was part of a national trauma, and these employees were part of a supportive and grieving Norwegian society (Thoresen, Aakvaag, Wentzel-Larsen, Dyb, & Hjemdal, 2012).

In line with studies showing that high perceived social support is associated with subsequently lower levels of posttraumatic stress (Freedman et al., 2015; Kaniasty & Norris, 2008; Shallcross et al., 2016), we found that social support increased the rate of recovery from psychological distress. This is also consistent with studies exploring the role of social support in recovery from clinical depression and anxiety. For example, in a sample of psychiatric hospital patients, initial levels of social support predicted clinical improvement in depression when controlling for other important risk factors (Brugha et al., 1990; Brugha, Bebbington, Stretch, MacCarthy, & Wykes, 1997). Another study found that following an evidence-based intervention for anxiety, perceived availability of global social support was associated with a change in depression over time (Dour et al., 2014). Interventions that foster social connectedness and seeing oneself as a valued member of a given social group affect psychological distress and may shorten recovery time (Cruwys et al., 2013, 2014; Jones et al., 2012). Thus, for individuals reporting high levels of psychological distress, social support may facilitate mental health recovery.

We also found that social support from family and friends, and support from leaders were independently associated with trauma recovery. In line with this, a cross-sectional study found that social support outside the work context independently accounted for variance in well-being when work factors were taken into account (Stansfeld, Shipley, Head, Fuhrer, & Kivimaki, 2013). This finding may be explained by the differing measures of social support from the different support sources. It has also been demonstrated that the role and effect of social support on health and psychological well-being varies depending on the source of support (Li et al., 2014; Walen & Lachman, 2000). Thoits (2011) has argued that there are essentially two support categories: emotional sustenance and active coping assistance. Our measure of general social support may reflect emotional sustenance. In contrast, our measures of support from leaders and colleagues may more accurately reflect active coping assistance. These can overlap, which may also explain why we did not find an independent effect of colleague support. Both emotional sustenance and instrumental support may be important in recovery processes, and may reflect different mechanisms of social support’s influence on psychological health.

The primary strengths of this study include a longitudinal design, a relatively high response rate, and an appropriate number of participants with high levels of psychological distress. Some study limitations are also worth noting. First, whereas social support may consist of many aspects that change dynamically over time, our measure of social support was heterogeneous in content across source, and only social support perceived as positive was assessed. In addition, the absence of assessments of social support and psychological distress prior to or sooner than 10 months after the traumatic event makes it difficult to determine whether the event or prior symptoms influenced respondents’ levels of social support or psychological distress. Furthermore, our sample was exclusively government employees, which may limit generalizability, and use of only self-report may not objectively reflect available social support.

## 5. Conclusions

In conclusion, these results suggest that for those who are highly distressed following a traumatic event, both general social support and leader support may play a role in their recovery. One implication of these findings is that clinicians working with psychologically distressed individuals should make specific efforts to increase perceived social support such as family education, interpersonal skill training and/or environmental changes. Furthermore, organizations that promote awareness and practices to enhance social support between leaders and employees may also use these findings to facilitate recovery among employees who experience high levels of psychological distress. Further research should use prospective designs that measure social support prior to possible traumatic experiences to enhance our understanding of the associations between social support and psychological stress, and test whether interventions aimed at increasing social support from leaders may benefit clinically distressed individuals in the workforce.

## Highlights of the article

- High levels of social support is related to low levels of psychological distress.
- This study investigates whether high levels of social support is related to change in psychological distress over time.
- High social support is related to faster recovery from high psychological distress after trauma.
- Leader support is of importance beyond the general social support from family and friends.

## References

[CIT0001] Arnberg F. K., Hultman C. M., Michel P.-O., Lundin T. (2012). Social support moderates posttraumatic stress and general distress after disaster. *Journal of Traumatic Stress*.

[CIT0002] Brewin C., Andrews B., Valentine J. (2000). Meta-analysis of risk factors for posttraumatic stress disorder in trauma-exposed adults. *Journal of Consulting and Clinical Psychology*.

[CIT0003] Brugha T., Bebbington P., MacCarthy B., Sturt E., Wykes T., Potter J. (1990). Gender, social support and recovery from depressive disorders: A prospective clinical study. *Psychological Medicine*.

[CIT0004] Brugha T., Bebbington P. E., Stretch D. D., MacCarthy B., Wykes T. (1997). Predicting the short-term outcome of first episodes and recurrences of clinical depression: A prospective study of life events, difficulties, and social support networks. *The Journal of Clinical Psychiatry*.

[CIT0005] Cohen S., Wills T. A. (1985). Stress, social support, and the buffering hypothesis. *Psychological Bulletin*.

[CIT0006] Cruwys T., Dingle G. A., Haslam C., Haslam S. A., Jetten J., Morton T. A. (2013). Social group memberships protect against future depression, alleviate depression symptoms and prevent depression relapse. *Social Science & Medicine*.

[CIT0007] Cruwys T., Haslam S. A., Dingle G. A., Jetten J., Hornsey M. J., Chong E. D., Oei T. P. (2014). Feeling connected again: Interventions that increase social identification reduce depression symptoms in community and clinical settings. *Journal of Affective Disorders*.

[CIT0008] Curran P. J., Bauer D. J., Willoughby M. T. (2004). Testing main effects and interactions in latent curve analysis. *Psychological Methods*.

[CIT0009] Dallner M., Elo A-L., Gamberale F., Hottinen V., Knardahl S., Lindstrøm K., Ørhede E. (2000). *Validation of the General Nordic Questionnaire (QPSNordic) for Psychological and Social Factors at Work*.

[CIT0010] Dour H. J., Wiley J. F., Roy‐Byrne P., Stein M. B., Sullivan G., Sherbourne C. D., Craske M. G. (2014). Perceived social support mediates anxiety and depressive symptom changes following primary care intervention. *Depression and Anxiety*.

[CIT0011] Duncan T. E., Duncan S. C., Strycker L. A. (2013). *An introduction to latent variable growth curve modeling: Concepts, issues, and application*.

[CIT0012] Elklit A., Pedersen S. S., Jind L. (2001). The Crisis Support Scale: Psychometric qualities and further validation. *Personality and Individual Differences*.

[CIT0013] Finne L. B., Christensen J. O., Knardahl S., Langguth B. (2014). Psychological and social work factors as predictors of mental distress: A prospective study. *Plos One*.

[CIT0014] Freedman S. A., Gilad M., Ankri Y., Roziner I., Shalev A. Y. (2015). Social relationship satisfaction and PTSD: Which is the chicken and which is the egg?. *European Journal of Psychotraumatology*.

[CIT0015] Graham J. W. (2009). Missing data analysis: Making it work in the real world. *Annual Review of Psychology*.

[CIT0016] Hansen M. B., Birkeland M., Nissen A., Blix I., Solberg Ø., Heir T. (in press). Prevalence and course of symptom-defined PTSD in individuals directly or indirectly exposed to terror - A longitudinal study. *Psychiatry*.

[CIT0017] Hansen M. B., Nissen A., Heir T. (2013). Proximity to terror and post-traumatic stress: A follow-up survey of governmental employees after the 2011 Oslo bombing attack. *BMJ Open*.

[CIT0018] House J. S., Kahn R. L., McLeod J. D., Williams D. (1985). Measures and concepts of social support. *Social support and health*.

[CIT0019] Hu L., Bentler P. M. (1999). Cutoff criteria for fit indexes in covariance structure analysis: Conventional criteria versus new alternatives. *Structural Equation Modeling: A Multidisciplinary Journal*.

[CIT0020] Jones J. M., Williams W. H., Jetten J., Haslam S. A., Harris A., Gleibs I. H. (2012). The role of psychological symptoms and social group memberships in the development of post-traumatic stress after traumatic injury. *British Journal of Health Psychology*.

[CIT0021] Kaniasty K., Norris F. H. (2008). Longitudinal linkages between perceived social support and posttraumatic stress symptoms: Sequential roles of social causation and social selection. *Journal of Traumatic Stress*.

[CIT0022] Kenny D. A., Kaniskan B., McCoach D. B. (2014). The performance of RMSEA in models with small degrees of freedom. *Sociological Methods & Research, 44,* 486–507.

[CIT0023] Kievit R. A., Frankenhuis W. E., Waldorp L. J., Borsboom D. (2013). Simpson’s paradox in psychological science: A practical guide. *Frontiers in Psychology*.

[CIT0024] King D. W., Taft C., King L. A., Hammond C., Stone E. R. (2006). Directionality of the association between social support and posttraumatic stress disorder: A longitudinal investigation1. *Journal of Applied Social Psychology*.

[CIT0025] Kline R. (2011). *Principles and practice of structural equation modeling*.

[CIT0026] Li B., Ma H., Guo Y., Xu F., Yu F., Zhou Z. (2014). Positive psychological capital: A new approach to social support and subjective well-being. *Social Behavior and Personality: an International Journal*.

[CIT0027] Little T. D. (2013). *Longitudinal structural equation modeling*.

[CIT0028] Lui A., Glynn S., Shetty V. (2009). The interplay of perceived social support and posttraumatic psychological distress following orofacial injury. *The Journal of Nervous and Mental Disease*.

[CIT0029] Marchand A., Blanc M.-È. (2011). Occupation, work organisation conditions and the development of chronic psychological distress. *Work*.

[CIT0030] Marchand A., Demers A., Durand P. (2005). Do occupation and work conditions really matter? A longitudinal analysis of psychological distress experiences among Canadian workers. *Sociology of Health & Illness*.

[CIT0031] Marchand A., Demers A., Durand P. (2006). Social structures, agent personality and workers’ mental health: A longitudinal analysis of the specific role of occupation and of workplace constraints-resources on psychological distress in the Canadian workforce. *Human Relations*.

[CIT0032] May C. L., Wisco B. E. (2015). Defining trauma: How level of exposure and proximity affect risk for posttraumatic stress disorder.

[CIT0033] Muthén B., Muthén L. K. (1998–2014). *Mplus (Version 7.2)*.

[CIT0034] Neria Y., DiGrande L., Adams B. G. (2011). Posttraumatic stress disorder following the September 11, 2001, terrorist attacks: A review of the literature among highly exposed populations. *American Psychologist*.

[CIT0035] Nielsen M. B., Tvedt S. D., Matthiesen S. B. (2012). Prevalence and occupational predictors of psychological distress in the offshore petroleum industry: A prospective study. *International Archives of Occupational and Environmental Health*.

[CIT0036] Ozer E. J., Best S. R., Lipsey T. L., Weiss D. S. (2003). Predictors of posttraumatic stress disorder and symptoms in adults: A meta-analysis. *Psychological Bulletin*.

[CIT0037] Pietrzak R. H., Johnson D. C., Goldstein M. B., Malley J. C., Southwick S. M. (2009). Psychological resilience and postdeployment social support protect against traumatic stress and depressive symptoms in soldiers returning from Operations Enduring Freedom and Iraqi Freedom. *Depression and Anxiety*.

[CIT0038] Price M., Evans M., Bagrow J. (2014). PTSD symptoms, disability, and social support in the acute period after a traumatic injury: A preliminary investigation of competing hypotheses. *Journal Trauma Stress Disor Treatment 4*.

[CIT0039] Rudenstine S., Galea S., Lindert J., Levav I. (2015). Terrorism and its impact on mental health. *Violence and mental health*.

[CIT0040] Santiago P. N., Ursano R. J., Gray C. L., Pynoos R. S., Spiegel D., Lewis-Fernandez R., Fullerton C. S. (2013). A systematic review of PTSD prevalence and trajectories in DSM-5 defined trauma exposed populations: Intentional and non-intentional traumatic events. *Plos One*.

[CIT0041] Santini Z. I., Koyanagi A., Tyrovolas S., Mason C., Haro J. M. (2015). The association between social relationships and depression: A systematic review. *Journal of Affective Disorders*.

[CIT0042] Schlenger W. E., Caddell J. M., Ebert L., Jordan B. K., Rourke K. M., Wilson D., Kulka R. A. (2002). Psychological reactions to terrorist attacks: Findings from the National Study of Americans’ Reactions to September 11. *Jama*.

[CIT0043] Shallcross S. L., Arbisi P. A., Polusny M. A., Kramer M. D., Erbes C. R. (2016). Social causation versus social erosion: Comparisons of causal models for relations between support and PTSD symptoms. *Journal of Traumatic Stress*.

[CIT0044] Stansfeld S. A., Shipley M. J., Head J., Fuhrer R., Kivimaki M., Dowd J. B. (2013). Work characteristics and personal social support as determinants of subjective well-being. *Plos One*.

[CIT0045] Strand B. H., Dalgard O. S., Tambs K., Rognerud M. (2003). Measuring the mental health status of the Norwegian population: A comparison of the instruments SCL-25, SCL-10, SCL-5 and MHI-5 (SF-36). *Nordic Journal of Psychiatry*.

[CIT0046] Thoits P. A. (2011). Mechanisms linking social ties and support to physical and mental health. *Journal of Health and Social Behavior*.

[CIT0047] Thoresen S., Aakvaag H. F., Wentzel-Larsen T., Dyb G., Hjemdal O. K. (2012). The day Norway cried: Proximity and distress in Norwegian citizens following the 22nd July 2011 terrorist attacks in Oslo and on Utøya Island. *European Journal of Psychotraumatology*.

[CIT0048] Walen H. R., Lachman M. E. (2000). Social support and strain from partner, family, and friends: costs and benefits for men and women in adulthood. *Journal of Social and Personal Relationships*.

[CIT0049] Wu K. K., Cheung M. W. (2006). Posttraumatic stress after a motor vehicle accident: A six month follow-up study utilizing latent growth modeling. *Journal of Traumatic Stress*.

